# *Aronia melanocarpa* Extract Ameliorates Hepatic Lipid Metabolism through PPARγ2 Downregulation

**DOI:** 10.1371/journal.pone.0169685

**Published:** 2017-01-12

**Authors:** Chung-Hwa Park, Jung-Hee Kim, Eun Byul Lee, Wonhee Hur, Oh-Joo Kwon, Hyoung-Jin Park, Seung Kew Yoon

**Affiliations:** 1 Division of Gastroenterology & Hepatology, Department of Internal Medicine, College of Medicine, The Catholic University of Korea, Seoul, Korea; 2 The Catholic University Liver Research Center (CULRC), The Catholic University of Korea, Seoul, Korea; 3 WHO Collaborating Center of Viral Hepatitis, The Catholic University of Korea, Seoul, Korea; 4 Department of Medical Biochemistry, College of Medicine, The Catholic University of Korea, Seoul, Korea; 5 MushMed Co., LTD., Chuncheon-si, Gangwon-do, Korea; Beckman Research Institute, UNITED STATES

## Abstract

Nonalcoholic fatty liver disease (NAFLD) is a hepatic manifestation of metabolic syndrome. Studies have demonstrated that anthocyanin-rich foods may improve hyperlipidemia and ameliorate hepatic steatosis. Here, effects of *Aronia melanocarpa* (AM), known to be rich of anthocyanins, on hepatic lipid metabolism and adipogenic genes were determined. AM was treated to C57BL/6N mice fed with high fat diet (HFD) or to FL83B cells treated with free fatty acid (FFA). Changes in levels of lipids, enzymes and hormones were observed, and expressions of adipogenic genes involved in hepatic lipid metabolism were detected by PCR, Western blotting and luciferase assay. In mice, AM significantly reduced the body and liver weight, lipid accumulation in the liver, and levels of biochemical markers such as fatty acid synthase, hepatic triglyceride and leptin. Serum transaminases, indicators for hepatocyte injury, were also suppressed, while superoxide dismutase activity and liver antioxidant capacity were significantly increased. In FL83B cells, AM significantly reduced FFA-induced lipid droplet accumulation. Protein synthesis of an adipogenic transcription factor, peroxisome proliferator-activated receptor γ2 (PPARγ2) was inhibited *in vivo*. Furthermore, transcriptional activity of PPARγ2 was down-regulated *in vitro*, and mRNA expression of PPARγ2 and its downstream target genes, adipocyte protein 2 and lipoprotein lipase were down-regulated by AM both *in vitro* and *in vivo*. These results show beneficial effects of AM against hepatic lipid accumulation through the inhibition of PPARγ2 expression along with improvements in body weight, liver functions, lipid profiles and antioxidant capacity suggesting the potential therapeutic efficacy of AM on NAFLD.

## Introduction

Nowadays, metabolic syndrome, and its associated conditions are becoming of medical interest [1–3]. Nonalcoholic fatty liver disease (NAFLD) is a hepatic manifestation of metabolic syndrome, characterized by impaired metabolic regulation in adipose tissue leading to expanded visceral fat accumulation, high serum triglyceride (TG), insulin resistance and fat deposition in the liver [4–6]. It is defined as TG content over 5% of liver weight in patients without significant alcohol consumption or any secondary causes for lipid accumulation in the liver. Accumulation of hepatic TG leading to dysregulation of hepatic lipid homeostasis is known to be the major pathophysiology of NAFLD [1, 6–8].

Lipid metabolism in the liver can be largely categorized into 1) free fatty acid (FFA) uptake, 2) lipogenesis and 3) oxidation of lipids, and derangement in any step will lead to NAFLD [9]. Hepatic steatosis is coordinated by transcriptional factors such as peroxisome proliferator-activated receptor γ (PPARγ), sterol regulatory element-binding protein 1c (SREBP1c) and carbohydrate-responsive element-binding protein (ChREBP) [9, 10]. PPARγ, activated as PPARγ-retinoid x receptor functional heterodimer, contributes to FFAs uptake and hepatic steatosis through PPARγ-responsive genes, such as lipoprotein lipase (LPL), fatty acid translocase, fatty acid transport proteins and adipocyte protein 2 (aP2) [11–14]. During early phase of adipocyte differentiation, aP2, a fatty acid binding protein, induced by PPARγ facilitates lipid transportation and coordinates lipid responses in cells [15, 16], and LPL catalyzes hydrolysis of TG in the circulating lipoproteins into FFAs and 2-monoacylglycerol during delivery of lipids to tissues [17]. In response to insulin, activated SREBP1c induces *de novo* lipogenesis to generate FFA in the liver catalyzed by fatty acid synthase (FAS) [9, 13, 18–20], and ChREBP acts together with SREBP1c to stimulate lipogenic genes in response to dietary carbohydrates [19, 21]. Furthermore, insulin resistance induces adipocyte lipolysis resulting in further increase of serum FFAs, which influx to the liver becoming an important source of TG [2, 8]. Increased intrahepatic TG during these processes is stored in lipid droplets which are intracellular organelles storing neutral lipids within cells [8]. On the other hand, PPARα is pivotal in mitochondrial, peroxisomal and microsomal FFA oxidation by inducing genes involved in FFA oxidation [9, 18, 22]. Oxidation of FFAs within mitochondria facilitates degradation of FFAs to acetyl-CoA in turn preventing hepatic lipid accumulation, while, when mitochondrial oxidation is impaired and FFAs accumulate in the cytosol as in insulin resistance, FFAs are alternatively oxidized by the peroxisomes and endoplasmic reticulum inducing reactive oxygen species (ROS), ER stress and lipid peroxidation leading to hepatocyte injury [9, 18, 19]. Therefore, imbalance of lipid metabolism and lipogenic gene expressions will consequently induce both excessive FFA accumulation and oxidative stress in the liver leading to either apoptosis or necrosis of hepatocytes resulting in hepatic lipotoxicity and subsequent progression to nonalcoholic steatohepatitis (NASH) [6, 18–20, 23].

Up to date, effective pharmacological treatment for NAFLD is unavailable, and lifestyle modifications including physical activity, weight control and improvements in diet are mostly recommended to delay the progression of metabolic syndrome and to improve liver histology [3, 7, 24]. In this regard, dietary components have been under study, and some bioactive compounds such as anthocyanins have been pointed out [24–27]. Anthocyanins are plant polyphenols determining the colors of fruits, vegetables, beans and cereals depending on the pH [28]. Recent studies demonstrated that anthocyanin-rich foods show powerful antioxidant, anti-inflammatory, anti-adipogenic and anti-carcinogenic properties [24–27, 29–31]. *Aronia melanocarpa* (AM), the black chokeberry, is a fruit recently in interest for being rich of anthocyanins [25]. In previous studies, AM reduced epididymal fat accumulation, improved lipid profiles and memory function, reduced chemical-induced liver injury, diminished inflammation and lipid peroxidation in rodents [26, 32–37], and also reduced waist circumferences with improving lipid profiles in human.[38, 39]. Nonetheless, its effect on hepatic lipid metabolism is less investigated. Therefore, we examined the effect of AM on hepatic lipid metabolism *in vivo* and *in vitro*.

## Methods and Materials

### Compounds

Spray-dried ethanol extract of *Aronia melanocarpa* (AM) was purchased from Daesan Co. (Gyeonggi-do, Korea, S1 Table). Oleic acid and palmitic acid were blended in 2:1 as a FFA compound [40].

### Animal care and experimental protocol

Male 5 week-old C57BL/6N mice (SCL Inc., Hamamatsu, Japan) were housed under a 12-hr light/dark cycle at a temperature (21 ± 2°C) and humidity (60 ± 5%) controlled room. General health monitoring of all animals were performed every day. Criteria for the health monitoring include wound, bleeding, hair brilliance, nasal discharge, eye discharge, ear color, anal and genital discharge, general motor activity. Body weights of all animals were monitored two times a week. No animal became severely ill or died before the experimental endpoint. All animals were euthanized by cervical dislocation after anesthetization by intraperitoneal injection of urethane at a single dose of 1.5 g/kg body weight.

Animals were randomly assigned to three groups, i.e. normal chow diet (NCD) group, high fat diet (HFD) group and HFD with AM (HFD+AM) group (n = 10/group). NCD group was fed with normal chow (12 kcal% Lard; Purina, Jeollabuk-do, Korea), and HFD group with HFD (60 kcal% Lard; Research Diet Inc., New Brunswick, Canada, S2 Table). HFD+AM group was fed with HFD and AM powder dissolved in water (50 mg/kg daily) [33, 34, 41, 42]. The diets were given in the form of pellets *ad libitum*, and AM solution was supplied with oral zonde for 12 weeks. Mice had free access to diet and water throughout the experiment. All animal care and experimental protocols were conducted in accordance with the guidelines for the Care and Use of Laboratory Animals from the Research Supporting Center for Medical Science of the Catholic University of Korea, and were approved by the ethics committee of the College of Medicine, Catholic University of Korea.

### Histological examination

Liver tissues were fixed in 10% buffered formalin and embedded in paraffin for hematoxylin and eosin (H&E) stain.

### Biochemical assays

Concentrations of lipids, enzymes or hormone levels were determined using commercial assay kits under the manufacturer's instructions as follows: liver triglyceride (Biovision, San Francisco, CA, USA), liver superoxide dismutase (SOD) (Dojindo, Kumamoto, Japan), liver antioxidant capacity (Sigma-Aldrich, St. Louis, MO, USA), liver FAS (USCN Life Science Inc., Wuhan, China), serum alanine aminotransferase (ALT; Asanpharm, Seoul, Korea), serum aspartate aminotransferase (AST; Asanpharm) and serum leptin (IBL, Gunma, Japan). The liver antioxidant capacity assay kit was based on trolox equivalent antioxidant capacity (TEAC) assay method as reported elsewhere [43].

### Cell culture and nile-red staining

The immortalized mouse hepatocytes cell line, FL83B cells (American Type Culture Collection, Manassas, VA), was cultured in Ham's F-12K (Kaighn's) medium (Gibco-BRL, Grand Island, NY, USA) supplemented with 10% fetal bovine serum (FBS), 100 U/ml of penicillin, 100 ug/ml of streptomycin, and 1% HEPES at 37°C in a humidified incubator with 5% CO_2_.

Cultured FL83B cells were incubated in serum-free F-12K medium for 24 hr, and then intracellular lipid accumulation was induced by treatment with 0.5 mM of FFAs (oleic acid:palmitic acid, 2:1). Various concentrations of AM (40 and 80 ug/mL, S3 Table) were added to the medium right after FFA treatment. Twenty-four hr later, the cells were subjected to Nile-red staining to evaluate the changes of intracellular lipid contents. The cells were washed with ice-cold phosphate-buffered saline (PBS) and fixed with 4% paraformaldehyde for 5 min at room temperature. After washing with PBS again, the cells were stained with Nile-red (0.5 μg/mL) and 4′,6-diamidino-2-pheny-lindole (DAPI, 1 μg/mL) (Sigma-Aldrich). After staining, intracellular lipid droplets were quantified by measuring fluorescence with a microplate reader (Molecular Devices, Sunnyvale, CA, USA), and normalized to the cellular DAPI contents [40]. The distribution of lipid in cells was observed under an LSM 510 inverted laser-scanning confocal microscope (Carl Zeiss, Jena, Germany).

### RNA extraction and reverse transcription-polymerase chain reaction (RT-PCR)

Total RNAs were extracted with TRIzol reagent (Invitrogen, Waltham, MA, USA), and purified according to the manufacturer’s recommendations. The purified RNAs were reverse-transcribed to single-stranded cDNA using random primers with Improm II reverse transcriptase (Promega, Fitchburg, WI, USA), and then amplified by PCR. The forward and reverse primers for mouse genes are shown in Table 1. The PCR was programmed as follows: 10 min at 94°C, 30 cycles of 94°C for 30 sec, 55°C for 30 sec, 72°C for 45 sec, and 10 min incubation at 72°C. The products were separated on 1.5% agarose gels containing 0.5 mg/mL ethidium bromide. The nucleic acids were visualized under UV light by Gel-Doc CQ system (Bio-Rad, Vienna, Austria), and the band densities were analyzed by Multi Gauge V3.0 program (Fujifilm Life Science, Tokyo, Japan). The expression of β-actin was used as a loading control.

**Table 1:** Primer sequences used for RT-PCR.

Gene	Primers	Sequences (5' → 3')
Peroxisome proliferatior-activated receptor gamma 2	F	TTCGGAATCAGCTCTGTGGA
	R	CCATTGGGTCAGCTCTTGTG
Adipocyte protein 2	F	AGCATCATAACCCTAGATGG
	R	GAAGTCACGCCTTTCATAAC
Lipoprotein Lipase	F	TGCCGCTGTTTTGTTTTACC
	R	TCACAGTTTCTGCTCCCAGC
Sterol regulatory element-binding protein 1c	F	ACTGGACACAGCGGTTTTGA
	R	TGTCAGCAGCAGTGAGTCTG
Carbohydrate-responsive element-binding protein	F	CCAGCCTCAAGGTGAGCAAA
	R	CATGTCCCGCATCTGGTCA
Peroxisome proliferatior-activated receptor alpha	F	AAGAACCTGAGGAAGCCGTTCTGT
	R	AGCTTTGGGAAGAGGAAGGTGTCA

F, forward; R, reverse.

### Western blot analysis

Frozen liver tissues of each mouse were pulverized in liquid nitrogen, and lysed with PRO-PREP^TM^ Protein Extraction Solution (iNtRon BIOTECHNOLGY, Gyeonggi-do, Korea) containing protease inhibitors for 20 min on ice. The lysates were centrifuged at 1,300 rpm (20 min, 4°C), and the total protein concentration was determined by Bradford assay (Bio-Rad, Hercules, CA, USA). Protein samples were separated with 10% SDS-PAGE and transferred to nitrocellulose membranes (Whatman, Maidstone, Kent, UK). The membranes were blocked with 5% skim milk in Tris-buffered saline solution containing Tween-20 (Sigma-Aldrich), and then incubated overnight at 4°C with primary antibodies to PPARγ (1:1000; Santa Cruz sc-7196, CA, USA) and monoclonal mouse anti-β-actin (1:2500; Sigma-Aldrich A2228). Membranes were washed with Tris-buffered saline containing 0.05% Tween-20 and incubated with horseradish peroxidase–conjugated anti-rabbit secondary antibody (1:5000; Amersham Pharmacia Biotech NA934, Piscataway, NJ, USA). Protein bands were visualized using an enhanced chemiluminescence system (Amersham Pharmacia Biotech) according to the manufacturer's instructions. The band densities were quantified by Multi Gauge V3.0 program (Fujifilm Life Science), and normalized to the protein expression levels of β-actin.

### Luciferase reporter assay

The change of transcriptional activity of PPARγ was analyzed by PPARγ–derived firefly luciferase activity. The reporter construct containing PPARγ–derived firefly luciferase gene, pDR1, was kindly provided from professor Oh-Joo Kwon (The Catholic University of Korea, Seoul, Republic of Korea). FL83B cells were co-transfected with pDR1 and pRL-TK (Promega, Seattle, WA, USA) containing CMV promoter-controlled *Renilla* luciferase gene using fuGENE HD (Promega, Seattle, WA, USA), and incubated with serum free media for 24 hr. Then the cells were treated with FFA and AM as described above, and, another 24 hr later, the cells were washed, lysed, and assayed for luciferase activity using a Dual-Luciferase reporter assay system (Promega) according to the manufacturer’s instructions. The luciferase activity was measured using a Veritas microplate luminometer (Tuner Biosystems, Sunnyvale, CA). The firefly luminescence signal was normalized to the *Renilla* luminescence signal.

### siRNAs targeting PPARγ2

PPARγ2 expression was down-regulated by transient transfection with siRNAs targeting PPARγ2 (si-PPARγ2, BIONEER, Daejeon, Republic of Korea) in FL83B cells. The FL83B cells were plated at a density of 5x10^4^ cells per 12-well culture dishes. 24 hours later, the cells were transfected with 50 nM of si-PPARγ2 using jetPRIME transfection reagent (Polyplus-transfection Inc., New York, NY, USA) according to the manufacturer’s protocol, and then incubated in serum-free F-12K medium for 24 hr. Intracellular lipid accumulation was induced by treatment with 0.5 mM of FFAs. 80 μg/mL of AM was added to the medium right after FFA treatment. Another 24 hours later, the changed levels of intracellular lipid were evaluated by Nile-Red staining as described above.

### Statistical analysis

Results were mostly expressed as the mean ± SD. Anthropometric parameters and biochemical data were acquired from all the mice enrolled, while RT-PCR and Western blot analysis data were acquired by analyzing liver samples pooled from four mice per group. *In vitro* data were acquired from at least three different cell culture sets. Comparisons of means were made using Student’s *t*-test, and were considered significant when the *p* < .05. (*p < .05, ** *p* < .001).

## Results

### AM prevents HFD-induced intrahepatic lipid accumulation and weight gain

**Fig 1:**
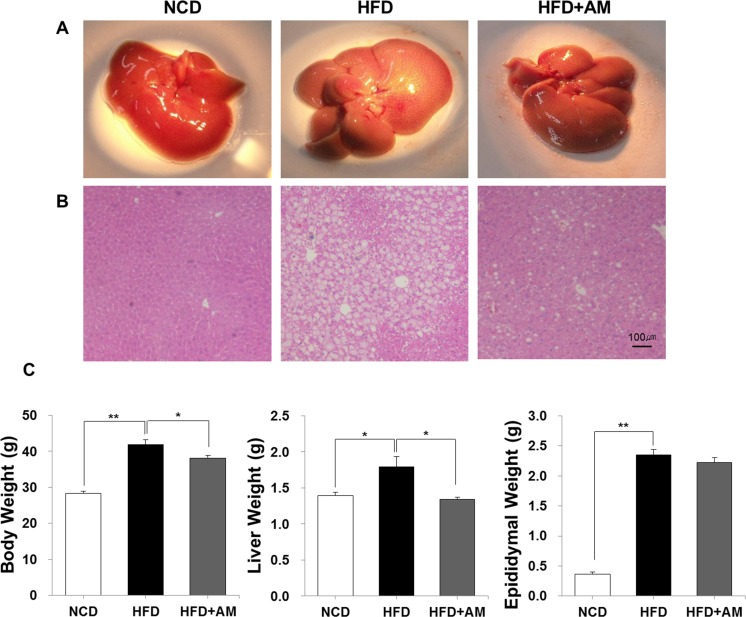
AM prevents HFD-induced intrahepatic lipid accumulation and weight gain. Hepatic steatosis was reduced in HFD+AM group both grossly (A) and histologically (B; H&E stain, magnification 100×). HFD-induced increase of body and liver weight were also significantly deterred by AM (C).

Grossly, the livers of HFD group were yellowish in color suggesting more lipid accumulation than those of NCD group, and, histologically, much lipid deposition was observed in HFD group (Fig 1A and 1B). Compared with HFD group, the livers of HFD+AM group were more pinkish, and lipid deposition was less observed histologically suggesting that AM restores the liver from HFD-induced lipid accumulation.

Body, liver and epididymal weights were measured (Fig 1C). Weight gain of body (*p* < 0.001), liver (*p* = 0.018) and epididymus (*p* < 0.001) was significant in HFD group. Mean body, liver and epididymal weight of NCD group were 28.37 ± 1.78 g, 1.39 ± 0.15 and 0.37 ± 0.10 g, and those of HFD group were 41.90 ± 4.17 g, 1.79 ± 0.43 g and 2.35 ± 0.28 g, respectively. HFD+AM group showed significantly less weight gain of body (HFD *vs* HFD+AM, 41.90 ± 4.17 *vs* 38.09 ± 2.24 g; *p* = 0.022) and liver (HFD *vs* HFD+AM, 1.79 ± 0.43 *vs* 1.34 ± 0.11 g; *p* = 0.005). Epididymal weight showed a slight decrease in HFD+AM group although not statistically significant (HFD *vs* HFD+AM, 2.35 ± 0.28 *vs* 2.22 ± 0.27 g; *p* = 0.421).

### AM diminishes HFD-induced increase of TG, FAS, hepatic enzymes and leptin

**Fig 2:**
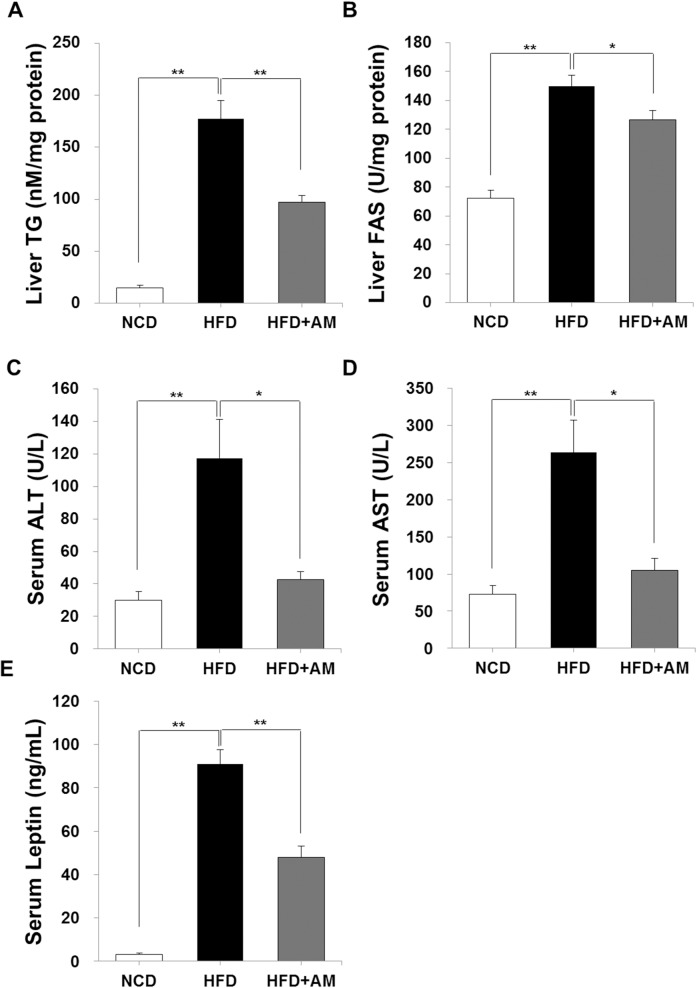
AM affects HFD-induced lipogenesis, hepatocellular injury and leptin level. While HFD induced significant elevation in intrahepatic TG (A), FAS (B), serum ALT (C), AST (D) and leptin (E), these were significantly inhibited in HFD+AM group.

In HFD group, hepatic TG and FAS were significantly elevated, while HFD-induced lipogenesis was inhibited by AM (Fig 2A and 2B). Mean hepatic TG and FAS levels in NCD group were 15.07 ± 7.84 nM/mg and 72.24 ± 17.22 U/mg protein, and those of HFD group were 177.20 ± 56.58 nM/mg and 149.80 ± 22.04 U/mg protein (both *p* < 0.001), respectively. In HFD+AM group, TG (97.35 ± 20.22 nM/mg, *p* < 0.001) and FAS (126.38 ± 21.45 U/mg protein, *p* = 0.043) was significantly less compared with HFD group.

Liver enzymes, ALT and AST, were elevated significantly in HFD group indicating that HFD has induced hepatocellular injury and inflammation. In NCD group and HFD group, serum ALT levels were 30.03 ± 16.26 U/L and 117.33 ± 71.26 U/L (*p* = 0.002), and serum AST levels were 72.50 ± 36.60 U/L and 263.59 ± 123.52 (*p* < 0.001), respectively. However, in HFD+AM group, serum ALT (42.76 ± 14.75 U/L, *p* = 0.012) and serum AST (105.01 ± 50.79 U/L, *p* = 0.003) levels were significantly lower showing preventive effect of AM on HFD-induced hepatocellular injury (Fig 2C and 2D).

Serum leptin level was also affected by HFD and AM (Fig 2E). Serum leptin levels of NCD, HFD, and HFD+AM group were 3.06 ± 1.94, 90.88 ± 21.53, and 47.83 ± 16.99 mg/mL, respectively (*p* < 0.001). Inhibitory effect of AM on HFD-induced serum leptin may indicate that AM has decreased body energy store.

### AM improves HFD-induced decrease of SOD and TEAC

**Fig 3:**
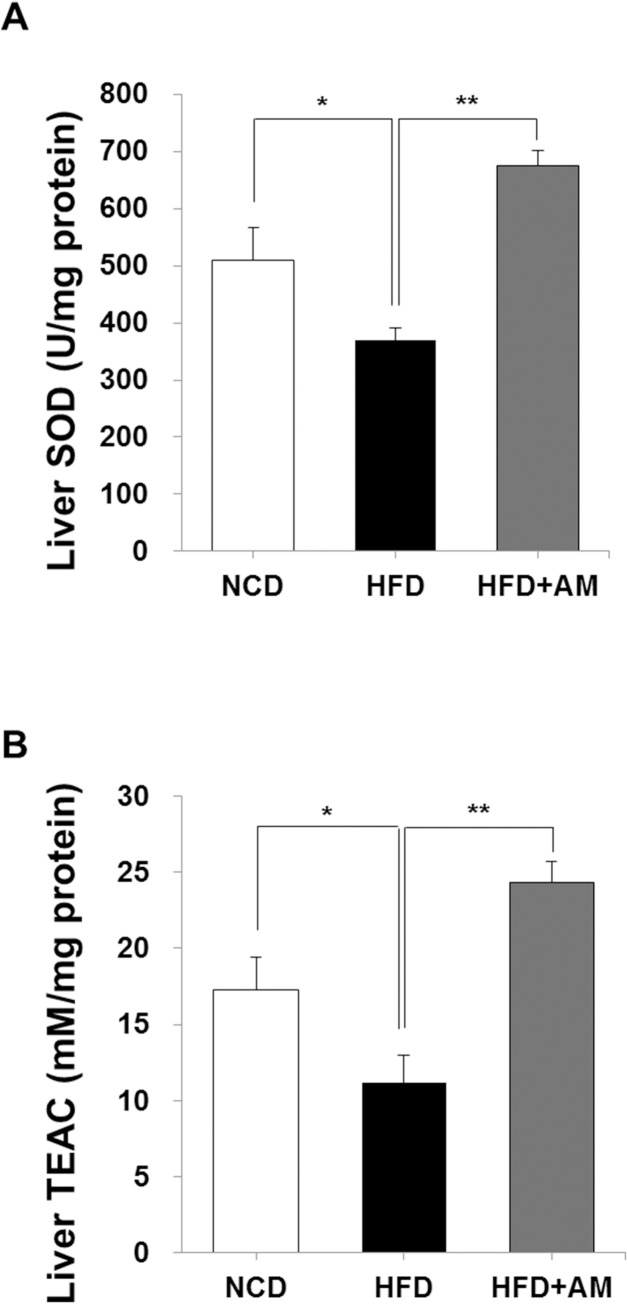
AM improves HFD-induced redox imbalance. Hepatic SOD activity (A) and TEAC (B) were decreased in HFD group, but these were significantly increased in HFD+AM group.

AM also showed protective effect on HFD-induced redox imbalance. In HFD group, SOD was significantly decreased (HFD *vs* NCD, 369.26 ± 68.29 and 509.11 ± 160.84 U/mg protein; *p* = 0.031), but SOD was significantly increased to 674.76 ± 82.08 U/mg protein in HFD+AM group (*p* < 0.001, Fig 3A).

Liver antioxidant capacity was analyzed to observe the changes of free radical scavenging activity by AM (Fig 3B). While liver TEAC was significantly decreased in HFD group (11.17 ± 4.84 mM/mg protein, *p* = 0.037) compared with NCD group (17.26 ± 5.98 mM/mg protein), it was significantly increased in HFD+AM (24.3 ± 4.35 mM/mg protein, *p* < 0.001).

### AM inhibits HFD-induced PPARγ2 *in vivo*

**Fig 4:**
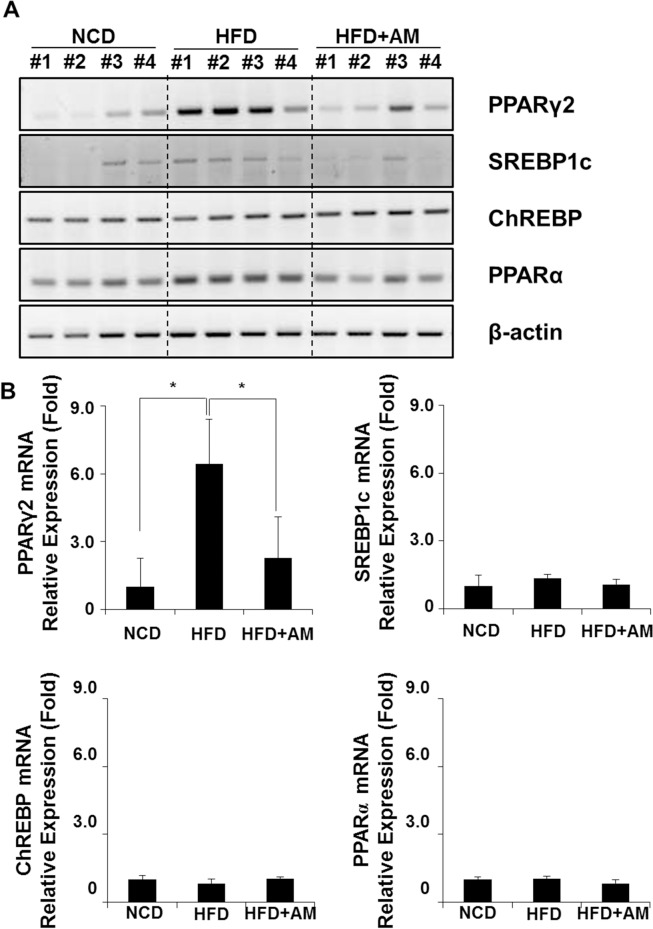
mRNA expressions of transcription factors related with hepatic lipid metabolsim were assessed. Changes of PPARγ2, SREBP1c, ChREBP and PPARα expressions from mice livers were analyzed by RT-PCR (A), and AM significantly inhibited HFD-induced PPARγ2 expression (B).

Expressions of major transcriptional factors involved in hepatic lipid metabolism, PPARγ2, SREBP1c, ChREBP and PPARα were screened by RT-PCR in mice livers (Fig 4, S1 Fig). Compared with NCD group, the mRNA expression of PPARγ2 was increased in HFD group, while it was attenuated in HFD+AM group (Fig 4A). Relative expression levels of each gene were analyzed by densitometry (Fig 4B). PPARγ2 in HFD group was over six-fold to that of NCD group (*p* = 0.004), and this was significantly decreased in HFD+AM group (*p* = 0.021). No significant differences were observed in SREBP1c, ChREBP and PPARα implying that AM affects the lipid metabolism through PPARγ2.

### PPARγ2 protein expression is affected by AM in vivo

**Fig 5:**
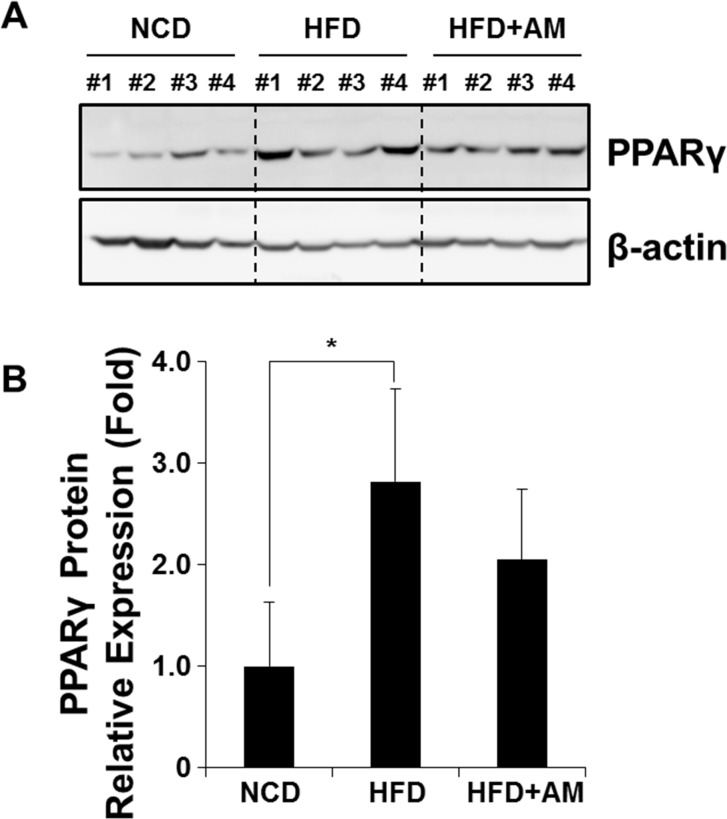
The changes in PPARγ2 protein expression were assessed in mice livers. Western blot analysis showed increase of PPARγ2 protein expression in HFD group and suppression of the protein in HFD+AM group (A) although the suppression was not statistically significant (B).

Western blot analyses were performed to assess whether the changes in PPARγ2 protein expression coincides with the changes in its mRNA expression in mice livers (Fig 5, S2 Fig). PPARγ2 protein was increased in HFD group compared with NCD group, while it was decreased in HFD+AM group (Fig 5A). Relative expression levels normalized by β-actin protein were also measured (Fig 5B). The increased expression of PPARγ2 protein by HFD was statistically significant (*p* = 0.017), and HFD+AM group showed decreasing tendency of PPARγ2 protein expression although the change was not statistically significant (*p* = 0.229).

### AM inhibits mRNA expressions of aP2 and LPL *in vivo*

**Fig 6:**
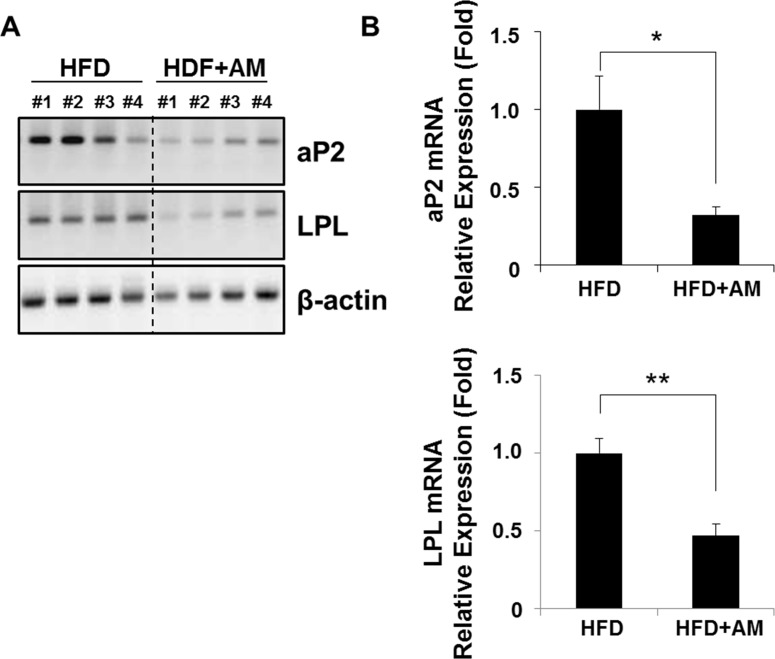
The expressions of aP2 and LPL mRNA were assessed in mice livers. aP2 and LPL mRNA showed decrease in HFD+AM group by RT-PCR (A), and both changes were statistically significant (B).

Since PPARγ2 mRNA was the most affected, downstream target genes of PPARγ2 such as aP2 and LPL were assessed in mice livers by RT-PCR (Fig 6, S3 Fig). The mRNA levels of aP2 and LPL were attenuated in HFD+AM group as expected (Fig 6A). The relative mRNA expression levels of aP2 (*p* = 0.022) and LPL (*p* < 0.001) genes decreased significantly in HFD+AM group (Fig 6B), indicating that AM ameliorates hepatic lipid metabolism through PPARγ2-dependent pathway.

### AM reduces FFA-induced intracellular lipid droplet accumulation

**Fig 7:**
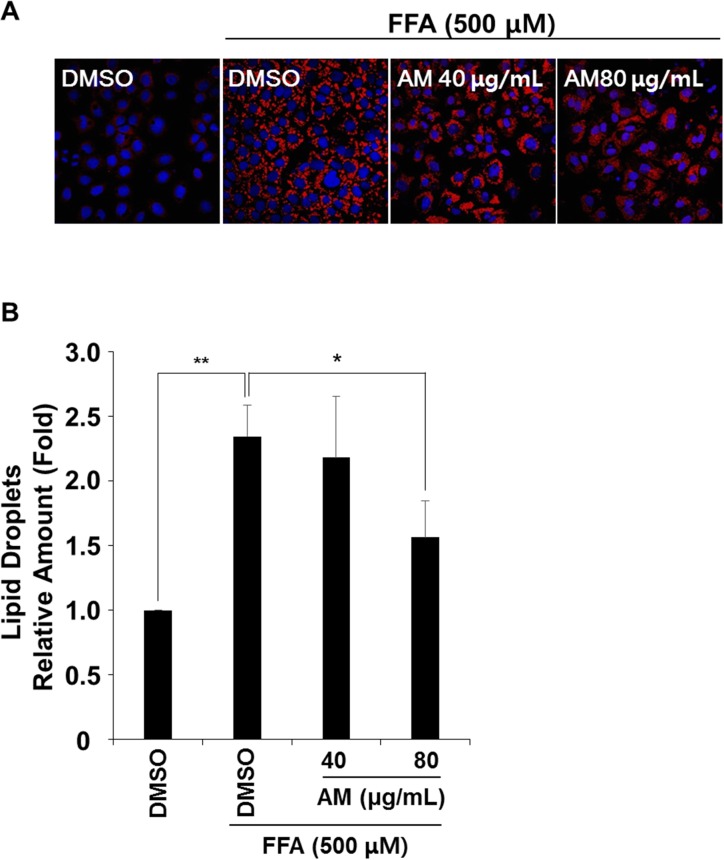
Nile-red stain was performed to show lipid droplets in FFA treated cells. FFA-induced lipid droplets were reduced in AM treated cells (bright red spots, A), and fluorometry revealed that AM reduced lipid droplets dose-dependently while FFA treatment increased lipid droplets over twofold of the control.

FL83B cells were treated with 0.5 mM FFA to induce lipid accumulation, and Nile-red staining was performed to identify the distribution and amount of intracellular lipid droplets. As shown by fluorescence microscopy (Fig 7A), intracellular lipid droplets (bright red spots) accumulated in 0.5 mM FFA-treated cell compared to mock, and AM treatment reduced lipid droplet accumulation dose-dependently. By fluorometry analysis (Fig 7B), 0.5 mM FFA treated cells showed significantly increased lipid accumulation by 2.35 ± 0.24 fold compared to mock (*p* = 0.001), which was inhibited dose dependently by AM treatment. In 40 ug/mL and 80 ug/mL of AM treated cells, lipid accumulation was 7% (*p* = 0.071) and 33.4% (*p* = 0.022) less than 0.5 mM FFA only treated cells.

### AM inhibits PPARγ2-dependent pathway *in vitro*

**Fig 8:**
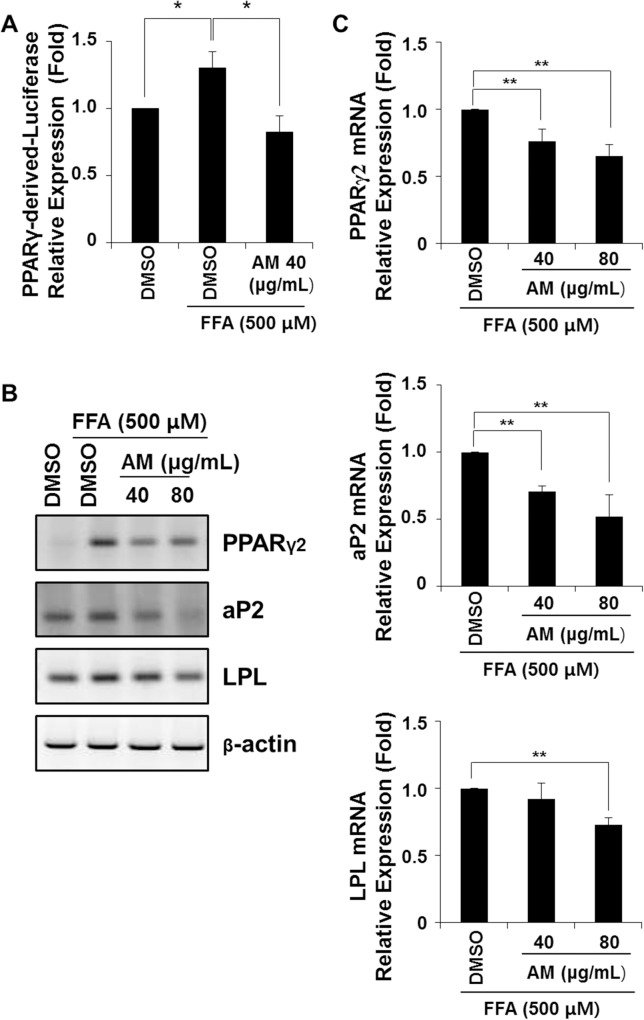
PPARγ2-depenent luciferase reporter assay was performed, and mRNA expressions of adipogenic genes in FL83B cell line were assessed by RT-PCR. The transcriptional activity of PPARγ2 was significantly decreased by AM treatment (A). PPARγ2, aP2 and LPL mRNA were increased by FFA, and reduced by AM (B), which were dose-dependent and statistically significant in all three genes (C).

To determine whether AM attenuates the transcriptional activity of PPARγ2 induced by FFA treatment, PPARγ2-depenent luciferase activities were analyzed in FL83B cells (Fig 8A). 0.5 mM FFA treatment significantly enhanced PPARγ2 transcriptional activity (*p* = 0.010), while this was significantly decreased in AM treated cells (*p* = 0.007).

The mRNA expression of genes that showed significant changes in mice livers were again analyzed in FL83B cells by RT-PCR (S4 Fig). mRNA levels of PPARγ2, aP2 and LPL increased when lipid accumulation was induced by 0.5 mM FFA treatment, and were suppressed by AM (Fig 8B). In densitometric analysis, AM dose-dependently reduced PPARγ2 (both *p* < 0.001) and aP2 (both *p* < 0.001) mRNA expression (Fig 8C upper, middle). LPL mRNA expression also decreased dose dependently, and was significant in AM 80 ug/mL (*p* < 0.001, Fig 8C, lower).

**Fig 9:**
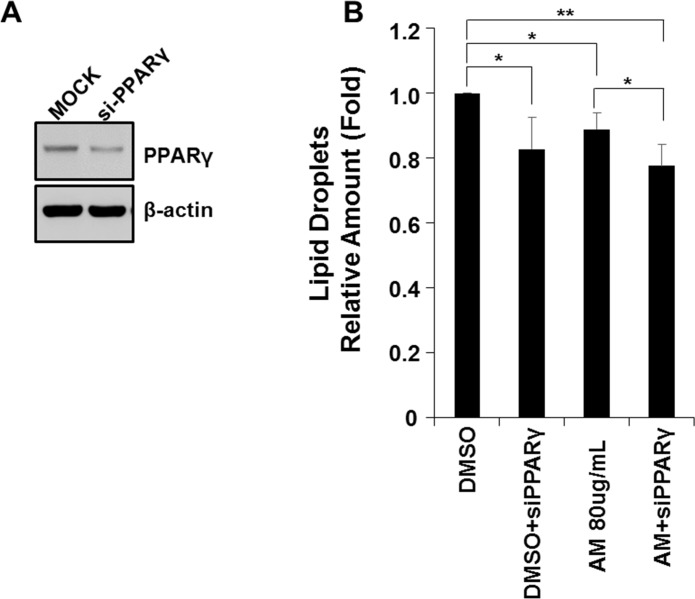
siRNA targeting PPARγ2 was used to silence the expression of PPARγ2 (A). The reduction of FFA-induced lipid accumulation by siRNA did not show significant difference from that of AM treated cells (B).

By silencing the expression of PPARγ2 using siRNA targeting PPARγ2, the effect of AM on transcriptional activity of PPARγ2 induced by FFA treatment was again determined (Fig 9). Transient transfection with siRNA targeting PPARγ2 in FL83B cells reduced FFA-induced lipid accumulation (*p* = 0.004). This reduction of FFA-induced lipid accumulation by siRNA was not significantly different from AM 80ug/mL treated cells (*p* = 0.245) or from siRNA and AM treated cells (*p* = 0.359). In FL83B cells treated with both siRNA and AM, FFA-induced lipid accumulation was more reduced compared with AM treated cells implying that siRNA has silenced the remaining PPARγ2 unaffected by AM (*p* = 0.016).

## Discussion

Since life style modification including fruit and vegetable enriched diet are emphasized in managing NAFLD, this study was focused on the effect of AM supplement in hepatic lipid metabolism. Major findings of this study was that intrahepatic lipid accumulation was hindered by AM, and this was mainly related to decreased expression of PPARγ2, a major adipogenic transcription factor, and its downstream target genes, aP2 and LPL.

First of all, AM supplement showed preventive effect in HFD-induced body and liver weight gain significantly, and lesser amount of lipid accumulation was visualized grossly and histologically (Fig 1). Liver plays a crucial role in lipid metabolism and homeostasis [1, 9]. Dysregulations in manufacturing, storing or exporting lipids occur during high caloric intake by increased hepatic uptake of FFAs from the serum stored as TG and by increased *de novo* FFA synthesis from acetyl-CoA by enhanced FAS in the liver [9, 18, 44]. During these processes, increased hepatic TG is stored as lipid droplets resulting in fatty liver [9]. In our data, along with anthropometric and histologic improvements, increase of hepatic TG during HFD was hindered by AM and FAS was less activated. These indicate that AM regulates HFD-induced *de novo* lipogenesis resulting in inhibition of hepatic TG accumulation.

Generally, elevated ALT and AST are well known markers for hepatocyte injury during inflammatory processes [2]. Serum ALT and AST elevations were also prevented by AM indicating that AM has protective effect on HFD-induced hepatocyte injury. NASH is characterized by inflammatory change accompanying steatosis, and is thought to be a more aggressive form of NAFLD [4]. Dietary intake of unsaturated fat increases plasma insulin levels resulting in insulin resistance, and subsequent inflammatory cascade induced by FFA oxidation and hepatic lipotoxicity play a role in progression from NAFLD to cirrhosis and HCC [6, 18–20, 23]. By hepatic steatosis, the redox potential of hepatocytes in both cytoplasm and mitochondria are changed toward a more reduced state [18]. This imbalance in hepatic redox system induces ROS production, and affects post-translational protein oxidation resulting in hepatocyte injury [9, 18–20, 23, 44]. Moreover, exercise and increase of energy expenditure, which are strongly recommended in metabolic syndrome, activate AMP-activated protein kinase which decreases lipid deposition in the liver but also stimulates lipid oxidation [1, 9]. Thus, enhanced SOD activity, increased TEAC and maintaining normal AST and ALT by AM collectively indicate that AM exerts antioxidant and anti-lipotoxic effects by modulating cellular redox environment, and that AM eventually prevents lipotoxicity and hepatocellular injury during HFD. This will delay the progression of NAFLD to NASH and hopefully to cirrhosis or HCC, and may also be beneficial in dealing with oxidative stresses during intensive physical activities to overcome obesity and fatty liver.

Leptin, the first described adipokine, is secreted proportionally to white adipose mass interfering with insulin signaling [9, 45, 46]. In hepatocytes, janus kinase 2/signal transducer and activator of transcription 3 pathway is the main pathway of its action, and leptin is involved in hepatic steatosis and insulin resistance through suppressor of cytokine signaling expression [45, 47]. When body energy store increases, circulating leptin level increases correlated with C-peptide, and plays a role in protecting the liver from lipotoxicity by inhibiting lipogenesis and glycogenolysis [1, 45, 46]. Here, serum leptin level was markedly elevated in HFD group, but significantly less increased in HFD+AM group implying that AM has diminished body energy store. Besides, lower serum leptin level may benefit not only in preventing insulin resistance and lipotoxicity, but also in restraining the progression of NASH by preventing fibrogenesis, since there are recent evidences that leptin acts as a profibrogenic cyokine in sinusoidal microenvironment by activating hepatic stellate cells to produce transforming growth factor β1 and other pro-inflammatory cytokines [1, 45, 48, 49].

Key transcription factors for hepatic lipid metabolism are PPARγ, SREBP1c, and ChREBP [9, 19]. PPARγ, the master transcription regulator of lipid metabolism, involves in steps such as induction of adipogenesis, preadipocyte differentiation, modification of lipoprotein metabolism and lipolysis by inducing proteins related to FFAs metabolism [11, 12, 50, 51], and its major function is FFA uptake and transport [9, 13, 14]. It exists in two isoforms, PPARγ 1 and 2, both excessive in hepatocytes contributing in fatty liver [52]. Primers for PPARγ2 mRNA were used to evaluate the effect of AM since it has been shown that high expression of PPARγ2 is positively associated with promoting lipid droplet formation both in human and rodents although only a small amount is expressed in normal liver [8, 10, 11, 51, 52]. It is also well known that the major function of SREBP1c is *de novo* lipogenesis in the liver through FAS in an insulin dependent manner, and that of ChREBP is *de novo* lipogenesis in response to dietary carbohydrate intake [9, 19]. PPARγ takes part in lipogenesis by regulating adipogenic genes including aP2, FAS and LPL [11, 13, 14, 26]. In this experiment, AM consumption in mice decreased hepatic PPARγ2, aP2 and LPL mRNA expression accompanied by decrease of PPARγ protein production while it did not affect SREBP1c and ChREBP. Also, AM significantly attenuated the FFA-induced mRNA expressions of PPARγ2 and its target genes, aP2 and LPL *in vitro*, and the transcriptional activity of PPARγ2. These were accompanied by decreased intracellular accumulation of lipid droplets. The effect of AM on transcriptional activity of PPARγ2 induced by FFA was once again determined by transfecting siRNA targeting PPARγ2 to FL83B cells. The effect of siRNA on FFA treated cells did not have significant difference compared with those treated with only AM or treated with both AM and siRNA. These results also support that AM has reduced FFA-induced lipid droplet accumulation through inhibition of PPARγ2 signaling in hepatocytes. In a previous report, AM up-regulated the mRNA expression of PPARγ in epididymal fat tissue, and reduced epididymal fat accumulation in rats [26]. Considering the physiologic function of PPARγ in white adipose tissue, i.e. improving insulin sensitivity [13, 14], these results indicate that AM may exert organ-specific activities, and that protective effect of AM against hepatic adipogenesis is by down-regulating the expression of PPARγ2 and its downstream events.

During the *in vivo* experiment, AM was orally administered. In general, anthocyanin is known as the most biologically active component in colored fruits and vegetables. AM is well known as an extremely rich source of anthocyanins, but also the least well absorbed by enteral feeding [25]. This study may not effectively support that anthocyanin was the active component to show the favorable effects. Nonetheless, our study adds evidence that the effect of AM can still be delivered orally effective enough to prevent hepatic FFA uptake and lipogenesis. These may also suggest that the purpose of fruit and vegetable rich diet in metabolic syndrome does not only mean high fiber diet to reduce caloric intake, but to gain antioxidative and anti-lipotoxic effects to prevent further progression and complications, even though dietary supplement of short-chain fatty acid, the main products of dietary fiber fermentation, obviously prevents and reverses HFD-induced metabolic abnormalities by decreasing PPARγ expression and activity [53]. To verify, further study on clinical application of AM remains.

In summary, the results show significant effect of AM on attenuating expressions of adipogenic genes, PPARγ2, LPL and aP2, in hepatocytes, and on inhibiting hepatic lipid accumulation with anti-oxidative properties. Furthermore, improvements in lipid profiles and liver function tests with lesser weight gain were obtained during AM consumption. Although there were efforts to show effects of AM on dysregulation of metabolic conditions, to our best knowledge, this is the first to show its effect through PPARγ2 related molecular pathway in reducing hepatic lipid accumulation. Taken together, we report a beneficial property of a natural product, AM, and its molecular mechanism in protecting hepatic lipid accumulation suggesting a new therapeutic application, hopefully as an oral agent, for managing NAFLD.

## Supporting Information

S1 Table Composition of phenolic compound in AM extract powder (provided by the manufacturer). (DOCX)

S2 Table Composition of HFD (60% kcal% fat diet, provided by the manufacturer). (DOCX)

S3 Table MTS assay. MTS assay was performed to assess cell viability within various conentraions of AM, and to determine AM concentrations for the experiment.
(DOCX)

S1 Fig Original pictures of Fig 4 showing RT-PCR results of PPARγ2, SREBP1c, ChREBP and PPARα mRNA from mice livers. (TIF)

S2 Fig Original pictures of Fig 5 showing Western blot results of PPARγ2 and β-actin expression from mice livers. (TIF)

S3 Fig Original pictures of Fig 6 showing RT-PCR results of aP2, LPL and and β-actin mRNA from mice livers. (TIF)

S4 Fig Original pictures of Fig 8B showing RT-PCR results of PPARγ2, aP2, LPL and β-actin mRNA in FL83B cell line. (TIF)

## Abbreviations

aP2: adipocyte protein 2
ALT: alanine aminotransferase
AM: *Aronia* *melanocarpa*
AST: aspartate aminotransferase
ChREBP: carbohydrate-responsive element-binding protein
DAPI: 4′,6-diamidino-2-pheny-lindole
FAS: fatty acid synthase
FFA: free fatty acid
H&E: hematoxylin and eosin
HFD: high fat diet
LPL: lipoprotein lipase
NAFLD: nonalcoholic fatty liver disease
NASH: nonalcoholic steatohepatitis
NCD: normal chow diet
PPAR: peroxisome proliferator-activated receptor
PBS: phosphate-buffered saline
ROS: reactive oxygen species
RT-PCR: reverse transcription-polymerase chain reaction
SREBP1c: sterol regulatory element-binding protein 1c
SOD: superoxide dismutase
TG: triglyceride
TEAC: trolox equivalent antioxidant capacity
