# Supplementary material for: Aronia melanocarpa Extract Ameliorates Hepatic Lipid Metabolism through PPARγ2 Downregulation
Source: PLoS One. 2017 Jan 12;12(1):e0169685. doi: 10.1371/journal.pone.0169685 (PMC5230775; doi:10.1371/journal.pone.0169685)
Supplement: S1 Table — (DOCX) [file pone.0169685.s001.docx]

**S1 Table. Composition of phenolic compound in AM extract powder (provided by the manufacturer).**

| Polyphenol | content (%) |
| --- | --- |
| Chlorogenic  acid | 6.20 |
| Neo Chlorogenic  acid | 6.01 |
| Flavanols | 1.96 |
| Cyanidin 3-galactoside | 14.89 |
| Cyanidin-3-glucoside | 0.89 |
| Cyanidin-3-arabinoside | 5.68 |
| Cyanidin-3-xyloside | 1.24 |
| Procyanidins | 40.1 |

Phenolic compounds consist of 76.97% of total extract.
