# Supplementary material for: Aronia melanocarpa Extract Ameliorates Hepatic Lipid Metabolism through PPARγ2 Downregulation
Source: PLoS One. 2017 Jan 12;12(1):e0169685. doi: 10.1371/journal.pone.0169685 (PMC5230775; doi:10.1371/journal.pone.0169685)
Supplement: S2 Table — (DOCX) [file pone.0169685.s002.docx]

**S2 Table. Composition of HFD (60% kcal% fat diet, provided by the manufacturer).**

| Product #D12492 | g% | kcal% |
| --- | --- | --- |
| Protein | 26.2 | 20 |
| Carbohydrate | 26.3 | 20 |
| Fat | 34.9 | 60 |
| Total |  | 100 |
| kcal/g | 5.24 |  |
| Ingredient | g | kcal |
| Casein, 30Mesh | 200 | 800 |
| L-Custine | 3 | 12 |
| Corn starch | 0 | 0 |
| Malrodextrin 10 | 125 | 500 |
| Sucrose | 68.8 | 275.2 |
| Cellulose | 50 | 0 |
| Soybean oil | 25 | 225 |
| Lard* | 245 | 2205 |
| Mineral Mix SI0026 | 10 | 0 |
| DiCalcium phosphate | 13 | 0 |
| Carlcium carbnate | 2.2 | 0 |
| Potassium Citrate | 16.5 | 0 |
| Vitamin Mix | 10 | 40 |
| DiCalcium phosphate | 2 | 0 |
| FD&C Blue Dye #1 | 0.05 | 0 |
| Total | 773.85 | 4057 |

*Typical analysis of cholesterol in lard = 0.72mg/g
