# Supplementary material for: Aronia melanocarpa Extract Ameliorates Hepatic Lipid Metabolism through PPARγ2 Downregulation
Source: PLoS One. 2017 Jan 12;12(1):e0169685. doi: 10.1371/journal.pone.0169685 (PMC5230775; doi:10.1371/journal.pone.0169685)
Supplement: S3 Table — MTS assay was performed to assess cell viability within various conentraions of AM, and to determine AM concentrations for the experiment. (DOCX) [file pone.0169685.s003.docx]

**S3 Table. MTS assay.** MTS assay was performed to assess cell viability within various conentraions of AM, and to determine AM concentrations for the experiment.

| Samples | AM (μg/mL) | | | | | | | |
| --- | --- | --- | --- | --- | --- | --- | --- | --- |
|  | 0 | 2.5 | 5 | 10 | 20 | 40 | 80 | 160 |
| O.D 490 | 1.0972 | 1.1283 | 1.1333 | 0.9945 | 0.9248 | 0.7795 | 0.8056 | 1.1586 |
|  | 1.0966 | 1.1922 | 1.2239 | 1.1232 | 0.9676 | 0.8202 | 0.9368 | 1.1434 |
|  | 1.1271 | 1.3391 | 1.2408 | 1.086 | 0.9629 | 0.7461 | 0.9407 | 1.1774 |
| Average | 1.107 | 1.2199 | 1.1993 | 1.0679 | 0.9518 | 0.7819 | 0.8944 | 1.1598 |
| Relative value | 1 | 1.1 | 1.08 | 0.96 | 0.86 | 0.71 | 0.81 | 1.05 |

Data were double checked with microscopic observation due to the original color of AM powder.
