# Supplementary figures and images for: Aronia melanocarpa Extract Ameliorates Hepatic Lipid Metabolism through PPARγ2 Downregulation
Source: PLoS One. 2017 Jan 12;12(1):e0169685. doi: 10.1371/journal.pone.0169685 (PMC5230775; doi:10.1371/journal.pone.0169685)

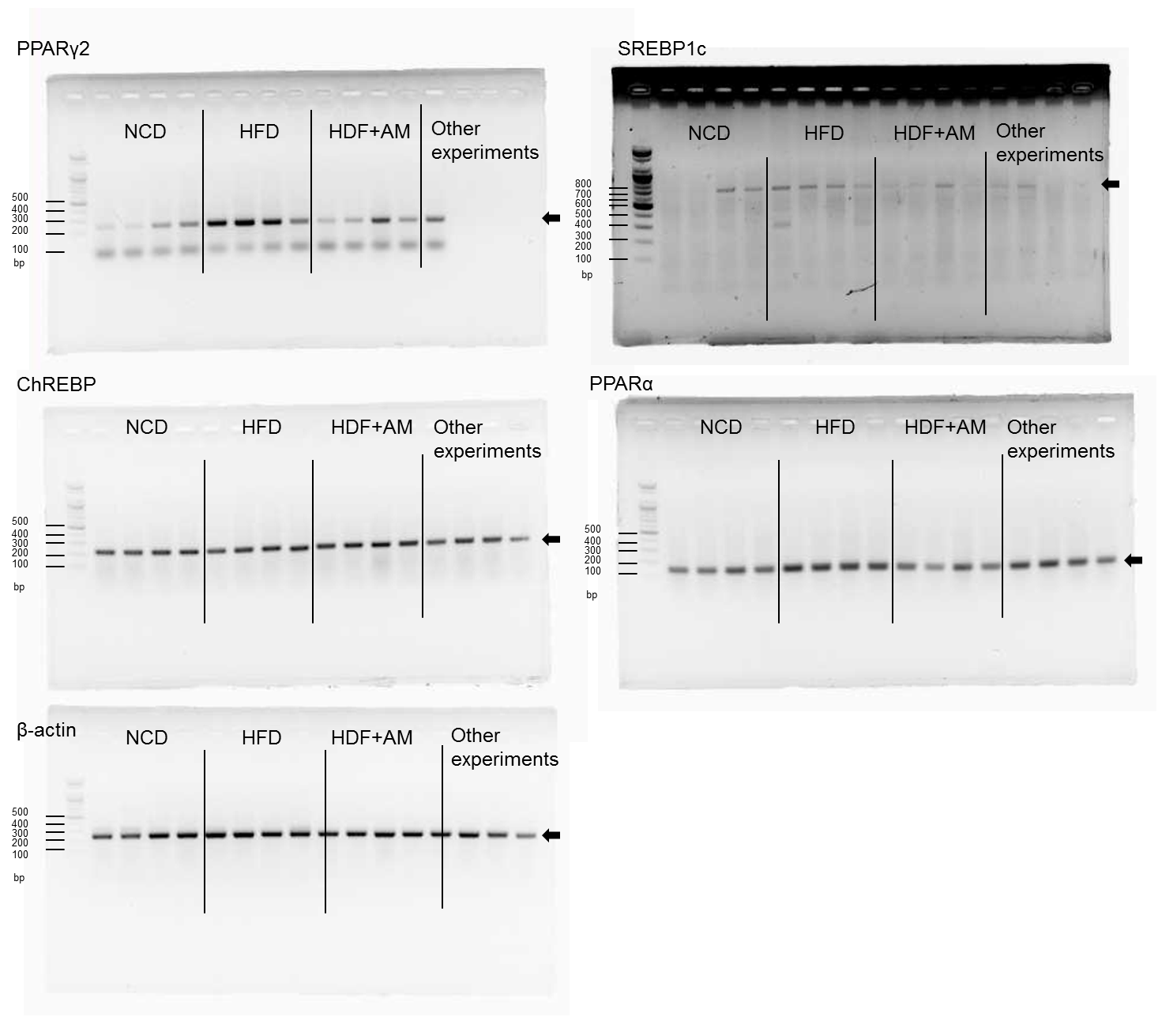

Supplement: S1 Fig — (TIF) [file pone.0169685.s004.tif]

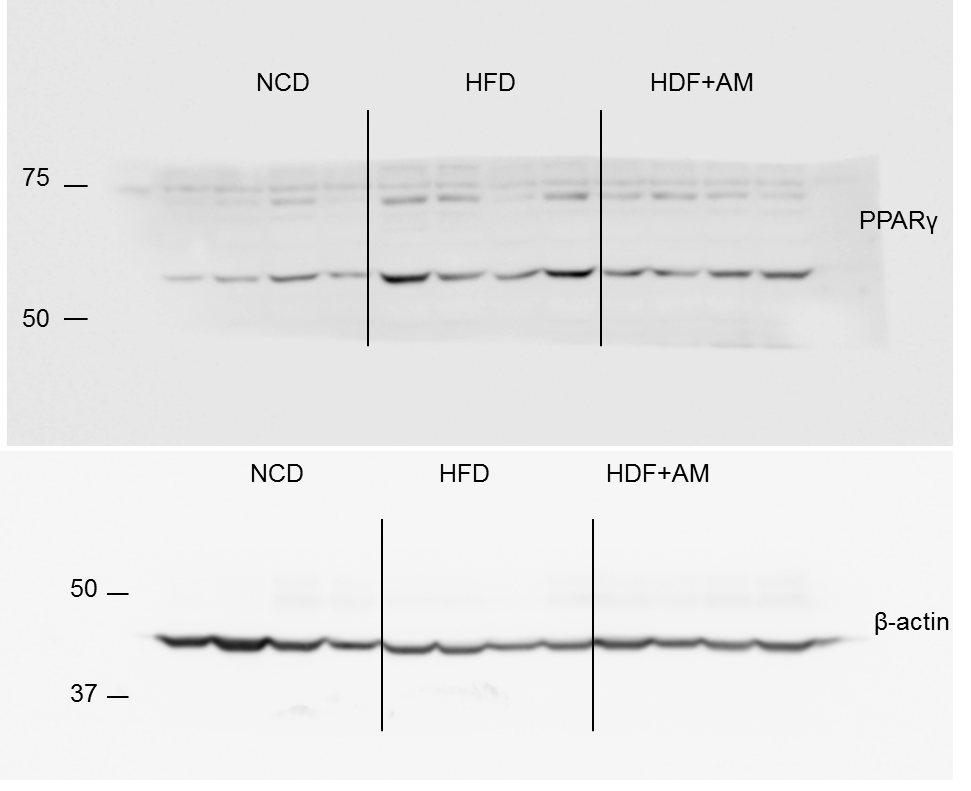

Supplement: S2 Fig — (TIF) [file pone.0169685.s005.tif]

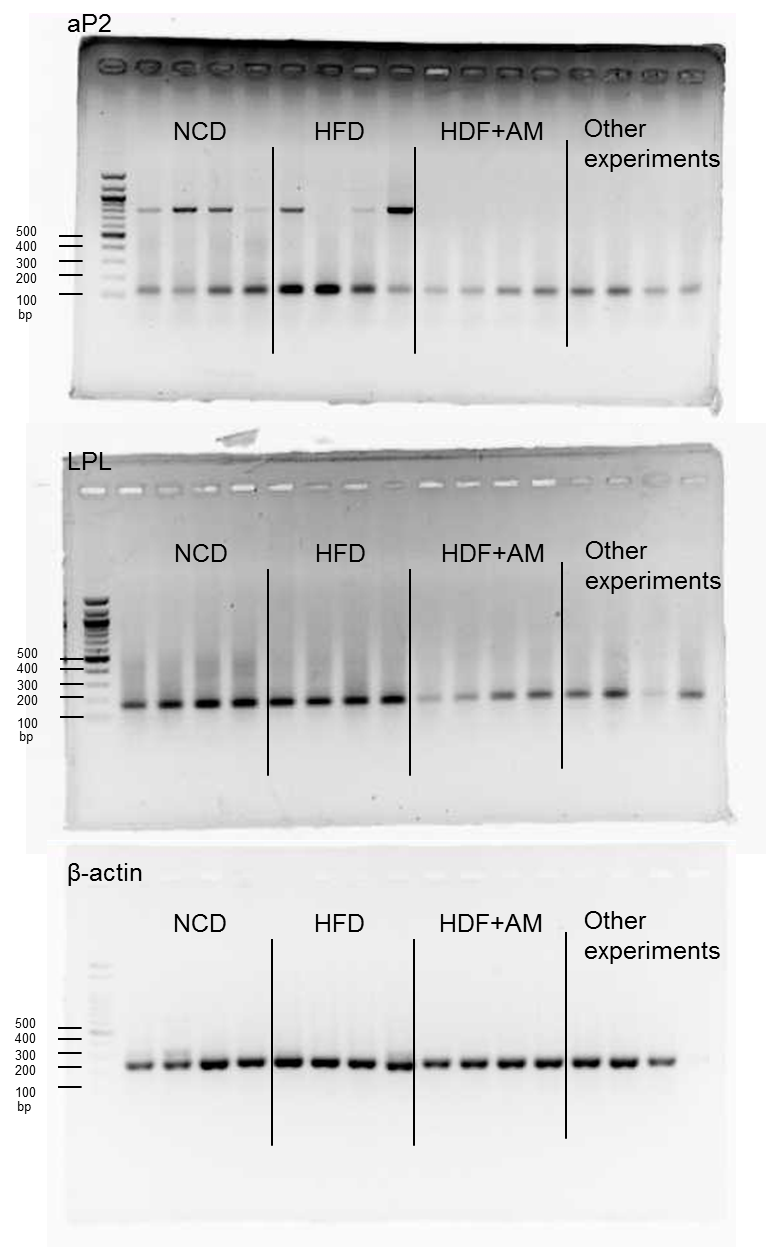

Supplement: S3 Fig — (TIF) [file pone.0169685.s006.tif]

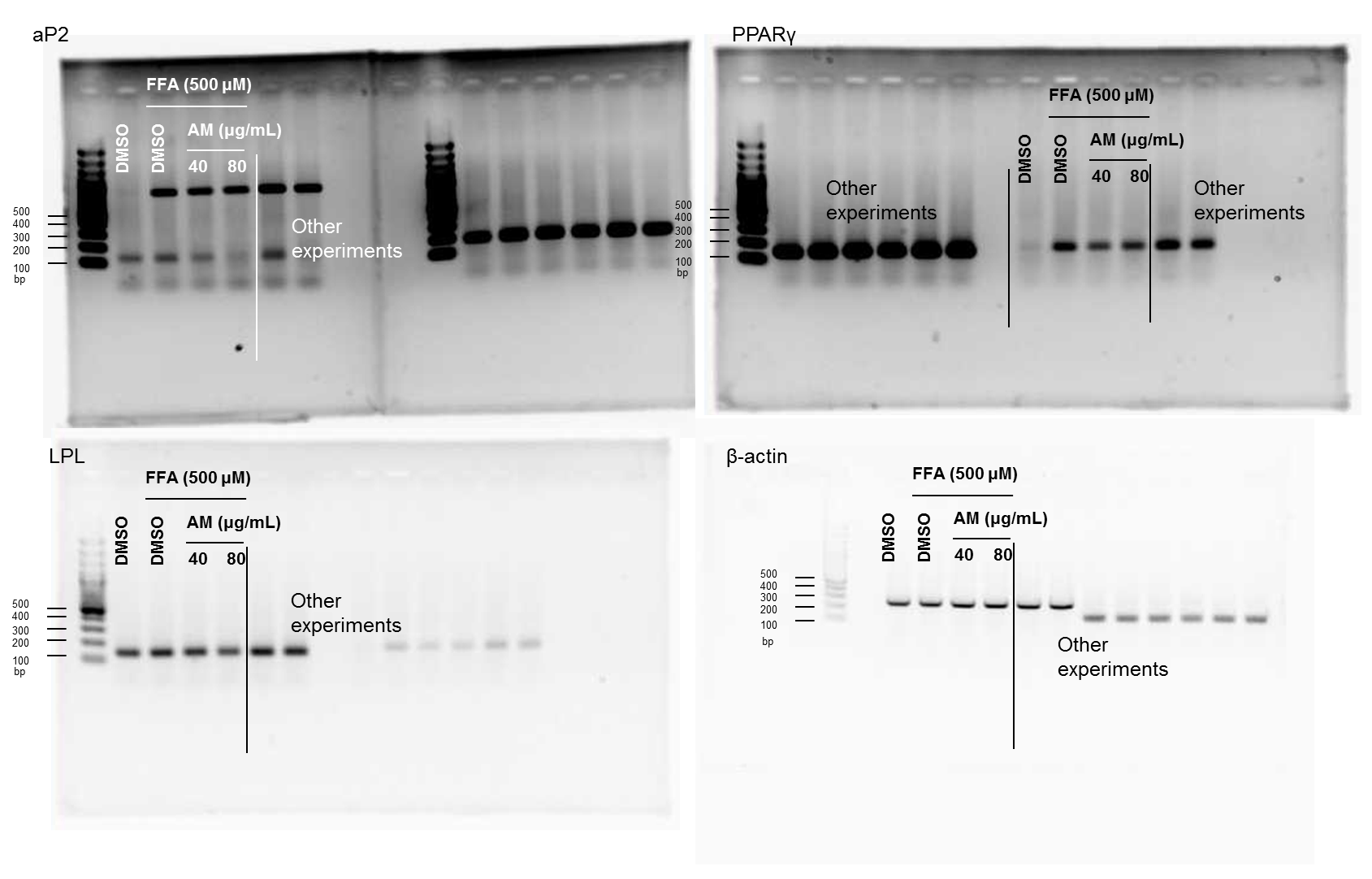

Supplement: S4 Fig — (TIF) [file pone.0169685.s007.tif]
